# On Slight Ailments; Their Nature and Treatment

**Published:** 1883-02

**Authors:** 


**On Slight Ailments; their Nature and Treatment.** By Leonel S. Beale, M. B., F. R. S., Fellow of the Royal College of Physicians, Professor of the Principles and Practice of Medicine in King's College, London; Physician to King's College Hospital, etc., etc. Second edition; enlarged and illustrated. Philadelphia: P. Blakiston, Son & Co., No. 1012 Walnut Street.

The first edition of this work was received with so much commendation, both from the medical press and practitioners, that it is hardly necessary to do more than announce that this second edition is now issued and that it contains all of the additions, illustrations and a complete index as in the last London edition. We venture to say that there are few books which will prove more useful to the young general practitioner, for whom it is more especially intended, than this able, practical work of Dr. Beale.

